# Technical nuances and approach-related morbidity of anterolateral and posterolateral lumbar corpectomy approaches—a systematic review of the literature

**DOI:** 10.1007/s00701-022-05240-8

**Published:** 2022-06-11

**Authors:** Christoph Wipplinger, Sara Lener, Christoph Orban, Tamara M. Wipplinger, Anto Abramovic, Anna Lang, Sebastian Hartmann, Claudius Thomé

**Affiliations:** 1 Department of Neurosurgery, Medical University Innsbruck, Anichstrasse 35, 6020 Innsbruck, Austria; 2 Department of Neurosurgery, University Hospital Würzburg, Würzburg, Germany; 3 Department of Biobehavioral Sciences, Teachers College, Columbia University, New York, NY USA

**Keywords:** Lumbar corpectomy, Anterolateral lumbar corpectomy, Posterolateral lumbar corpectomy, Vertebral body resection

## Abstract

**Purpose:**

Approaches for lumbar corpectomies can be roughly categorized into anterolateral (AL) and posterolateral (PL) approaches. It remains controversial to date whether one approach is superior to the other, and no comparative studies exist for the two approaches for lumbar corpectomies.

**Methods:**

A systematic review of the literature was performed through a MEDLINE/PubMed search. Studies and case reports describing technique plus outcomes and possible complications were included. Thereafter, estimated blood loss (EBL), length of operation (LOO), utilized implants, neurological outcomes, complication rates, and reoperation rates were analyzed.

**Results:**

A total of 64 articles reporting on 702 patients including 513 AL and 189 PL corpectomies were included in this paper. All patients in the PL group were instrumented via the same approach used for corpectomy, while in the AL group the majority (68.3%) of authors described the use of an additional approach for instrumentation. The EBL was higher in the AL group (1393 ± 1341 ml vs. 982 ± 567 ml). The LOO also was higher in the AL group (317 ± 178 min vs. 258 ± 93 min). The complication rate (20.5% vs. 29.1%, *p* = 0.048) and the revision rate (3.1% vs. 9.5%, *p* = 0.004) were higher in the PL group. Neurological improvement rates were 43.8% (AL) vs. 39.2% (PL), and deterioration was only noted in the AL group (6.0%), while 50.2% (AL) and 60.8% (PL) showed no change from initial presentation to the last follow-up.

**Conclusion:**

While neurological outcomes of both approaches are comparable, the results of the present review demonstrated lower complication and revision rates in anterolateral corpectomies. Nevertheless, individual patient characteristics must be considered in decision-making.

## Introduction

Lumbar corpectomies represent one of the most challenging spinal procedures. Common indications for lumbar corpectomy include fractures due to trauma, osteoporosis, or neoplastic infiltration of vertebral bodies [23, 49, 63]. Despite the abundant availability of literature addressing the outcome of thoracolumbar corpectomies, only a few studies report isolated outcome data specifically for lumbar corpectomies [15, 22, 39, 42, 53, 61, 63, 69, 70, 80, 83, 88].

Due to the anatomical differences between the lumbar spine and thoracic spine, numerous distinctions between corpectomy approaches have been described. Biomechanically, the lumbar spine is more flexible than the thoracic spine. The rib cage makes the thoracic spine less mobile, and hence thoracic corpectomies require less instrumentation compared to lumbar corpectomies [57, 63]. Adjacent segment disease is less common in the thoracic spine. Moreover, proximal or distal junctional failure related to the apex of the thoracic kyphosis or the thoracolumbar junction may occur [33]. Additionally, approach-related obstacles such as abdominal organs, vasculature, lumbosacral plexus, and lumbar spinal nerve roots may pose challenges [79].

Lumbar corpectomy approaches may be categorized as anterolateral (AL) and posterolateral (PL). Traditionally, the AL approach was considered the gold standard for accessing the lumbar anterior column, as it provides direct visualization of the vertebral body [79, 84]. However, AL approaches may be less familiar to spine surgeons and frequently require additional posterior instrumentation in order to achieve a biomechanically stable fusion construct [49, 80]. Corpectomy via a PL approach was viewed as more challenging due to the lack of direct visualization of the pathology via a small transpedicular working channel. Moreover, the placement of a vertebral body replacement (VBR) device for anterior column reconstruction via a posterior route is complicated by the lumbar nerve roots that cannot be sacrificed, as it may be acceptable in the thoracic spine [36]. With recent technical advances such as expandable cages and neuronavigation, several limitations of the PL approach could be mitigated to a certain extent [33, 64]. Advantages of the PL approach include early visualization of neural structures as well as the possibility of transpedicular instrumentation in a single session.

The aim of the present review was to compare technical nuances and approach-related morbidity of the AL and the PL approach for lumbar corpectomy.

## Methods

A systematic review of articles reporting on lumbar corpectomies, including approach-related morbidity, technical details, and outcomes, was performed according to the preferred reporting items for systematic reviews and meta-analyses (PRISMA) statement. A primary source for the electronic database search was MEDLINE/PubMed, supplemented by Google Scholar and reference lists of reviewed articles. Search strings included “lumbar corpectomy” and “thoracolumbar corpectomy” combined with the terms “anterior,” “posterior,” and “lateral” as well as “anterolateral” and “posterolateral.” We screened articles published between January 1999 and December 2021.

**Fig. 1:**
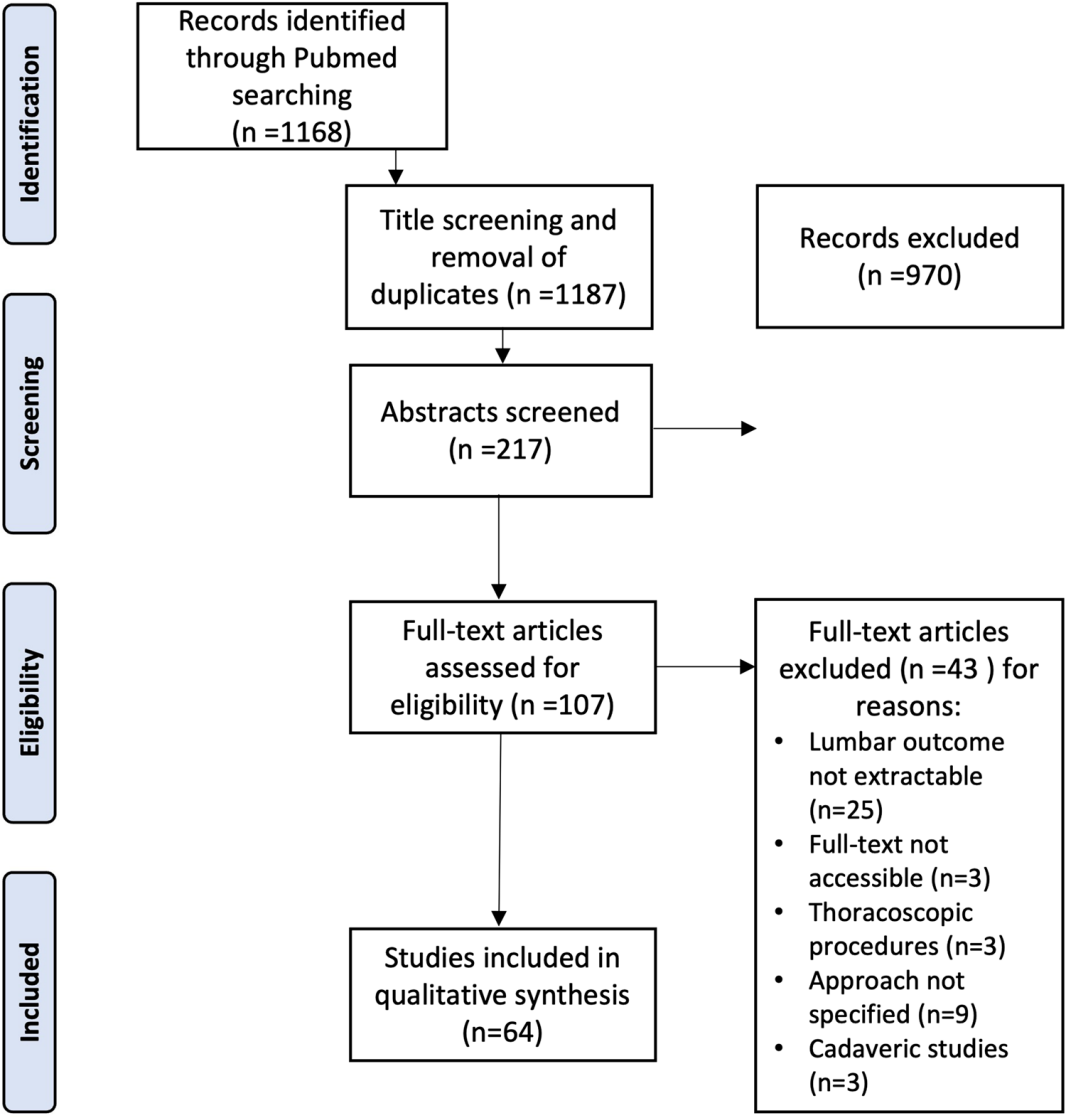
Screening algorithm

Retrospective and prospective studies as well as case reports were included if they reported clinical data as well as technical descriptions of AL and PL lumbar corpectomy approaches. Publications on thoracolumbar corpectomies were only included if outcome data of lumbar corpectomies were reported separately. A detailed description of our screening algorithm is provided in Fig. 1. To classify the level of evidence of the included studies, we adhered to the guidelines by Rutka (2017). Studies and patients were divided into AL and PL approaches and evaluated separately. The number of resected vertebral bodies and extent of instrumented fusion as well as the device used for VBR and the underlying pathology leading to the corpectomy procedure were assessed. For a more detailed analysis of interbody fusion, we evaluated if the instrumentation was performed over the same or an additional posterior approach in the case of an AL corpectomy.

### Statistical analysis

For the statistical analysis, we separated the collected data into an AL and a PL group. We analyzed estimated blood loss (EBL), length of operation (LOO), and rates of neurological improvement and deterioration, as well as reoperation rates due to approach-related complications of each study. Rates were stated in percentages. The entire analysis was based on the number of patients included in the articles, in which the respective parameters were reported. We used the Mann–Whitney *U* test to compare the means of continuous variables (EBL and LOO). For categorical variables (complications and outcomes), the chi-square test was performed. A *p*-value of < 0.05 was considered statistically significant.

## Results

### Included studies

Sixty-four articles reporting a total of 702 patients were included in this systematic review. Anterolateral corpectomies were described in 41 articles, PL in 25, including two articles reporting on both AL and PL corpectomies. The latter two were included in both groups as they provided a separate analysis of patients. The included articles consisted of 38 retrospective studies, two prospective studies, and 24 case reports. There were 36 evidence level IV studies, three level III (one study PL only, two studies both AL and PL) studies, and one level II study (PL only) (Table 1). Among the included articles, 17 reported exclusively on lumbar corpectomies. In the remaining 47 studies, information about lumbar corpectomy patients was either provided separately or the necessary data was easily extractable.

**Table 1:** Overview of included studies; *y* yes, *n* no, *AL* anterolateral, *PL* posterolateral, *I* infection, *T* trauma, *D* degenerative and deformity, *N* neoplasia, *O* osteoporosis, *VBR* vertebral body replacement, *SC* static cage, *EC* expandable cage, *AB* autologous bone only, *BC* bone cement, *N/A* not available, *EBL* estimated blood loss, *ST* surgical time, *NI* neurological improvement, *ND* neurological deterioration, *NU* neurologically unchanged

Author	Year	Level of evidence	Lumbar only	Number of patients	Approach	Pathology	VBR	Instrumentation approach	Fused vertebrae	EBL	ST	Complications	Revsion surgeries	NI	ND	NU
Alhashash et al. [2]	2021	IV	n	33	AL	D, O	EC	Additional	3	N/A	N/A	N/A	N/A	N/A	N/A	N/A
Alshareef et al. [3]	2020	IV	n	9	PL	N	EC	Same	4	N/A	N/A	N/A	N/A	N/A	N/A	N/A
Aryan et al. [5]	2007	IV	n	4	AL	I	SC, EC	Additional	N/A	N/A	N/A	1	0	N/A	N/A	N/A
Bhat et al. [6]	1999	IV	n	7	AL	T (5), I (2)	SC	Same	3	N/A	N/A	N/A	N/A	2	0	5
Byvaltsev et al. [7]	2021	IV	n	16	AL	T	EC	Additional	3	N/A	N/A	N/A	N/A	N/A	N/A	N/A
Cho [9]	2017	IV	n	3	PL	D	SC	Same	5	550	201	N/A	N/A	2	0	1
Choi et al. [10]	2017	IV	n	11	PL	T	EC	Same	3	1007	208.8	2	0	8	0	3
Chou et Lu [11]	2011	III	n	3	PL	T (2), I (1)	EC	Same	N/A	1067	560	2	1	0	0	3
Dai [15]	2010	IV	y	15	AL	N (6), I (9)	EC, AB	Same	N/A	N/A	N/A	1	0	9	0	6
Dvorak et al. [20]	2003	IV	n	30	AL	D (4), N (2), T (25), I (1)	EC	N/A	3	N/A	N/A	1	1	N/A	N/A	N/A
Elnady et al. [22]	2017	IV	y	17	PL	N (9), T (3), I (5)	SC, EC	Same	5	744	186.1	2	1	N/A	N/A	N/A
Gezercan et al. [27]	2016	IV	n	7	PL	N	EC	Same	5	N/A	N/A	N/A	N/A	4	0	3
Grobost et al. [28]	2020	IV	n	33	AL	T	EC	Additional	N/A	N/A	N/A	N/A	N/A	N/A	N/A	N/A
Hofstetter et al. [33]	2011	IV	n	17	PL	T	EC	Same	3	1150	324	3	3	N/A	N/A	N/A
Jandial et al. [39]	2013	IV	y	11	PL	N	EC	Same	5	1618	393	5	2	6	0	5
Joubert et al. [41]	2015	IV	n	4	PL	N	EC	Same	5	862	164	N/A	N/A	N/A	0	N/A
Knoeller et al. [42]	2012	IV	y	45	AL	N	EC	Same	N/A	N/A	N/A	7	0	19	0	26
Koller et al. [43]	2021	IV	y	17	AL	D	EC	Additional	N/A	N/A	N/A	16	N/A	0	10	7
Korovessis et al. [44]	2008	IV	n	23	AL	I	SC	Additional	4	860	270	1	1	9	0	14
Liljenqvist et al. [48]	2003	IV	n	13	AL	I	SC	Additional	5	1965	340	1	0	1	2	10
Lu et al. [49]	2010	III	n	27	AL (24), PL (3)	N (8), T (12),I (7)	SC,EC,AB, BC	Both	3	N/A	N/A	10	0	8	2	17
Lu et al. [50]	2009	III	n	12	AL (10), PL (2)	I	SC,EC,AB	Both	N/A	N/A	N/A	1	1	8	0	4
Mallepally et al. [62]	2021	IV	y	45	PL	T	SC	Same	4	1280	220	8	1	15	0	30
Mühlbauer et al. [53]	2000	IV	y	5	AL	O (4), N (1)	SC	Both	3	1120	360	1	0	5	0	0
Nakashima et al. [56]	2021	IV	n	25	AL	O	SC,EC	Additional	3	N/A	N/A	N/A	N/A	N/A	N/A	N/A
Payer et Sottas [58]	2008	IV	n	26	AL	N (5), T (21)	EC	Additional	3	670	191	3	0	N/A	N/A	N/A
Pham et al. [61]	2017	IV	y	7	PL	T	EC	Same	5	N/A	N/A	10	2	2	0	5
Richardson et al. [63]	2017	IV	y	42	AL	T	EC	Same	3	N/A	N/A	1	0	N/A	N/A	N/A
Sasani et Özir [64]	2009	IV	n	9	PL	T	EC	Same	3	467	182.2	N/A	N/A	3	0	6
Scheufler [65]	2007	IV	n	10	AL	N (7), T (1), I (2)	EC	Additional	N/A	420	175	6	1	5	0	4
Senel et al. [66]	2007	IV	n	3	PL	N	BC	Same	3	N/A	N/A	N/A	N/A	N/A	N/A	N/A
Shen et al. [67]	2008	IV	n	8	PL	N	EC	Same	4	1350	318	3	1	N/A	N/A	N/A
Shousha et al. [69]	2014	IV	y	25	AL	O (3), N (11), T (8), I (3)	EC	Both	3	3230	158	2	0	12	0	13
Singh et al. [70]	2012	IV	y	15	PL	N (12), I (3)	EC	Same	5	2400	N/A	N/A	N/A	3	0	12
Smith et al. [73]	2010	IV	n	34	AL	T	EC	Both	N/A	300	128	N/A	N/A	N/A	N/A	N/A
Street et al. [76]	2007	II	n	8	PL	N	BC	Same	N/A	N/A	N/A	5	3	N/A	N/A	N/A
Theologis et al. [80]	2016	IV	y	12	AL	T	EC	Additional	N/A	988	288.7	2	2	4	0	8
Vazan et al. [83]	2017	IV	y	14	AL	D(2), N(3), T(2), I(7)	SC, EC	Additional	4	N/A	186.9	4	3	N/A	N/A	N/A
Wong et al. [86]	2014	IV	n	1	PL	N	EC	Same	4	1000	360	N/A	N/A	N/A	N/A	N/A
Zidan et al. [88]	2019	IV	y	30	AL	O (4), N (8), T (12), I (6)	SC, EC, AB, BC	Additional	5	600	220	13	2	19	1	10
*Case reports* [4, 8, 12, 13, 16–19, 21, 24–26, 29, 35, 36, 52, 55, 68, 71, 75, 77, 78, 82]		*V*	*y*		*AL (n* = *27); PL (n* = *6)*	*D (4) T(2), I (4), N (14), O (3)*	*SC, EC, AB, BC*		*3*	*N/A*	*N/A*	*N/A*	*5*	*N/A*	*N/A*	*N/A*

### Indications

Data from 513 patients with AL lumbar corpectomies could be extracted. Indications included traumatic fractures in 223 patients (58.5%), tumors in 107 (28.1%), infections in 86 (22.6%), osteoporotic lesions in 70 (18.4%), and degenerative changes or deformity in 27 (7.1%) patients. Posterolateral lumbar corpectomy was reported in 189 patients, including 99 (73.9%) patients with trauma, 74 (55.2%) with neoplasia, 12 (9.0%) with infection, three (1.6%) with degenerative changes or deformity, and one patient (0.5%) with osteoporosis (Table 2).

**Table 2:** Indications for corpectomy

	Anterolateral		Posterolateral	
	*n*	%	*n*	%
Trauma	223	58.53%	99	52.38%
Neoplasia	107	28.08%	74	39.15%
Infection	86	22.57%	12	6.35%
Osteoporosis	70	18.37%	1	0.53%
Degeneration and deformity	27	7.09%	3	1.59%
Total	**513**		**189**	

### Procedures

Overall, a median of 1 (1–2) vertebral bodies was resected per patient. The majority of articles reported the use of expandable cages (AL 61.0%; PL 64.0%) and static cages (AL 14.6%; PL 16.0%) for VBR. The remaining articles described autologous bone only, bone cement, or a variety of the aforementioned VBR devices.

In the AL group, an additional approach for posterior transpedicular instrumentation was reported in 28 papers (68.3%), eight (19.5%) fused with unilateral anterior screw/plate constructs, and four (9.8%) used both anterior and posterior approach instrumentation, while information was not available in one (2.4%) article. In the PL group, a transpedicular instrumentation was performed in the same operative session in 23 (92.0%) papers, while one (4.0%) paper described the use of an additional approach, and one (4.0%) described both approaches. The majority of authors in the AL group (56.1%) described instrumentation one level above and below the corpectomy compared to the PL with two levels above and below instrumentation (64.0%) (Table 1).

The overall EBL and LOO averaged 1200 ± 1057 ml (data from 32 articles) and 301 ± 159 min (data from 27 articles) respectively. The EBL tended to be higher in the AL group (1393 ± 1341 ml) compared to the PL group (982 ± 567 ml, *p* = 0.787). The LOO turned out to be 317 ± 178 min in the AL group, and 258 ± 93 min in the PL group (*p* = 0.796).

Among papers reporting on AL corpectomies, a great heterogeneity regarding the technical nuances and access routes was noted. The choice for an access route was described based on the corpectomy level and the surgeon’s comfort as well as on the underlying pathology. Moreover, a clear time-based trend was observed. Earlier publications reported larger incisions, cylindrical cages or bone cement for VBR, and open pedicle screw instrumentation [6, 16, 20, 48, 55]. More recent articles tended to describe smaller openings, wide rectangular footplate cages, and percutaneous pedicle screw placement [1, 25, 56, 73, 80, 84].

### Complications

The 115 approach-related complications were reported in 34 articles including 499 patients (AL 351; PL 148), resulting in an overall complication rate of 32.8% (Table 3). In the PL group (29.1%), the complication rate was significantly higher than in the AL group (20.5%; *p* = 0.048).

**Table 3:** Approach-related complications reported in 34 articles including 499 patients, *p*-values < 0.05 were considered statistically significant

	Anterolateral (*n* = 351)		Posterolateral (*n* = 148)	
Wound infection/dehiscence	3.99%	14	2.70%	4
Durotomy	1.42%	5	10.14%	15
Pulmonary complications	2.28%	8	0.00%	0
Vascular injury	2.28%	8	0.00%	0
Post-OP hemorrhage	0.00%	0	2.70%	4
Implant-related	3.70%	13	8.11%	12
Organ/peritoneal injury	1.71%	6	0.00%	0
Nerve damage	3.42%	12	2.70%	4
Adjacent segment disease	1.71%	6	2.70%	4
Overall complication rate	**20.51%**		**29.05%**	

**Fig. 2:**
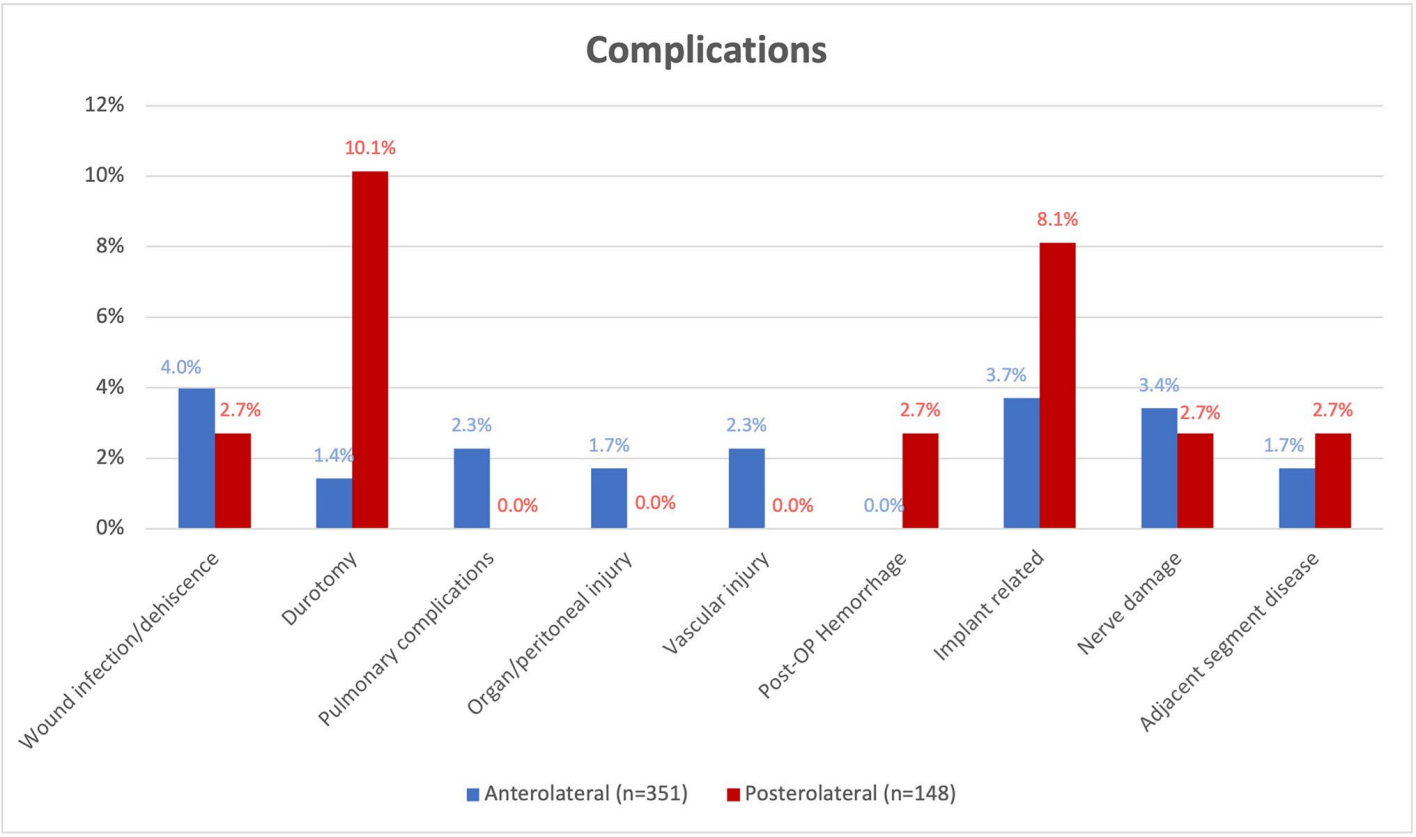
Approach-related complication rates in anterolateral (AL) and posterolateral (PL) corpectomies

Implant-related complications (PL 8.1% vs. 3.7%; *p* = 0.045) and durotomy (PL 10.1% vs AL 1.4% *p* < 0.001) were significantly more common in the PL group. Surgical wound problems (AL 4.0% vs. 2.7%, *p* = 0.610) and nerve damage (AL 3.4% vs 2.7% *p* = 0.797) occurred at slightly different rates in both groups. Adjacent segment disease was reported in 1.7% of articles in the AL group, and in 2.7% of those in the PL group, thus significantly more in the PL group (*p* = 0.492). Vascular injury (2.3%), organ damage (1.7%), and pulmonary complications (2.3%) were only described in the AL group. Postoperative hematoma (2.7%) was exclusively reported in the PL group (Fig. 2).

### Outcomes

**Fig. 3:**
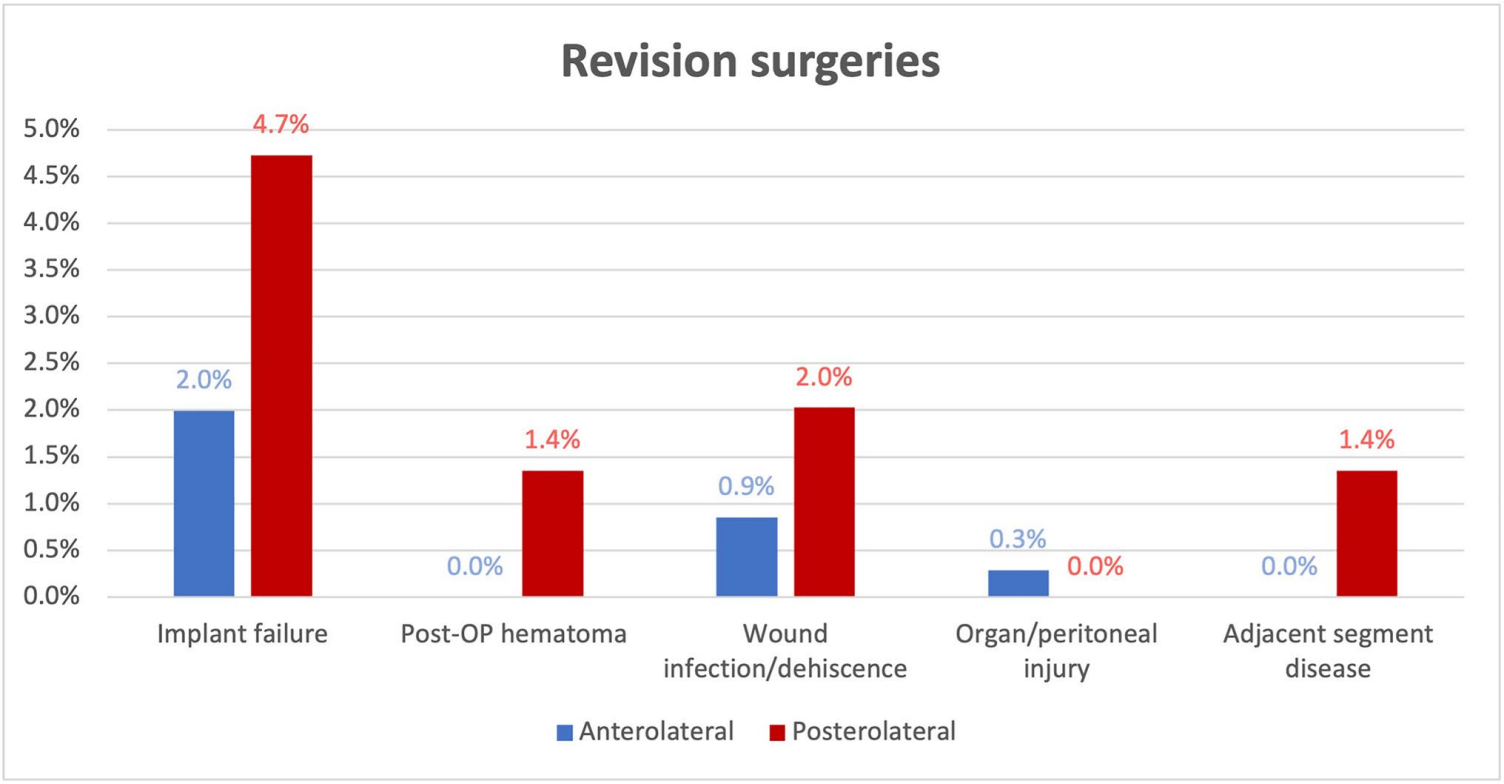
Revision surgeries due to complications from anterolateral (AL) and posterolateral (PL) corpectomies

A total of 25 patients required revision surgery for the abovementioned approach-related complications resulting in an overall revision rate of 5.0%. Revision surgery was significantly more frequently required in the PL group (9.5%) than in the AL group (3.1%, *p* = 0.004). Implant-related complications represented the most common reason for revision surgery (2.0% in the AL group, 4.7% in the PL group) (Fig. 3).

Neurological outcomes could be extracted from 39 studies with 371 patients (AL 251; PL 120).

**Fig. 4:**
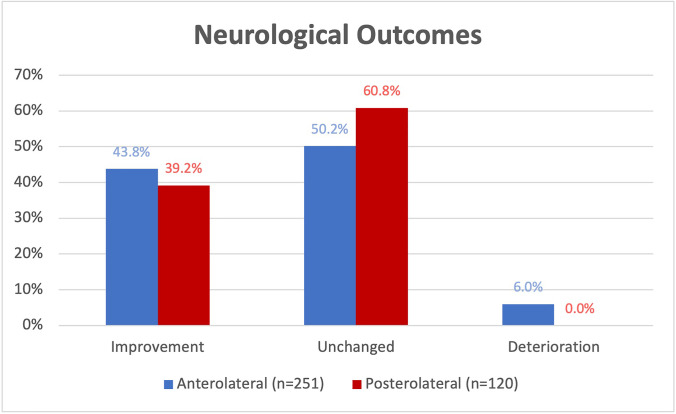
Neurological outcomes of anterolateral (AL) and posterolateral (PL) corpectomies

In the AL group, 43.8% of patients improved, 6.0% deteriorated, and 50.2% showed no change from initial presentation to the last follow-up. In the PL group, 39.2% of patients improved, 60.8% showed no change, and none of the patients deteriorated (*p* = 0.158) (Fig. 4).

## Discussion

To date, there are no studies comparing AL and PL approaches for lumbar corpectomies, and only few comparative studies for thoracic and thoracolumbar approaches exist [49, 85]. The main focus of this review was to analyze the AL and PL lumbar corpectomy approaches comparing technical issues, complications, and outcomes. One of the main challenges was to identify as many mutual parameters as possible to perform a direct comparison between the two approaches. The most common parameters outlined in the analyzed studies were represented by indications for surgery, procedure-related issues, perioperative complications, including revision surgery, as well as the clinical outcome.

### Indications

Overall, we identified traumatic burst fractures as the most frequent indication for both AL and PL corpectomies while neoplastic infiltration of the vertebral bodies represented the second most common indication. While technically more challenging and associated with higher complication rates, the posterior approach is more versatile. In more complicated traumatic lumbar fractures with extensive damage to both the anterior and the posterior column, both columns can be addressed through a single PL approach [61]. The same is true for neoplastic vertebral infiltrations. Most of these lesions are due to metastases of breast, lung, colorectal, prostate, and kidney tumors. These infiltrations are mainly located extradurally and predominantly affect the anterior part of the vertebral bodies [30]. Spinal cord compression as a result of epidural spread often leads to the first clinical symptoms in these patients, so that a posterior approach with decompression, tumor mass reduction, VBR devices, and instrumented fusion is indicated. If the surgical goal is gross total tumor resection, the removal of all affected parts up to a complete en bloc vertebrectomy followed by circumferential reconstruction of both columns can be performed via a single-stage PL approach [33, 39, 49, 67]. Lumbar corpectomy for spinal infections was more commonly addressed via an AL approach, as anterolateral access corridors allow for more radical debridement and drainage of frequently accompanying psoas abscesses under direct visualization [44, 47].

### Variations in access routes in relation to the corpectomy levels

Especially among AL approaches, several variations including a lateral retroperitoneal pre- or transpsoas approach, an anterior retroperitoneal or transperitoneal approach, and a lateral transdiaphragmatic approach to the thoracolumbar junction are performed. Since the upper, middle, and lower lumbar spine confront the surgeon with particular approach-related challenges, the choices for the abovementioned access routes were described depending on the corpectomy level. When accessing the upper lumbar vertebral bodies at the thoracolumbar junction, pleura, diaphragm, peritoneum, and overlying ribs are potential obstacles that need to be taken into consideration. While most authors described a partial rib resection, the greatest variation in thoracolumbar junction access corridors was found regarding the diaphragm. In general, the upper lumbar vertebrae can be accessed either via a craniocaudal-supradiaphragmatic or a caudocranial-infradiaphragmatic route [65]. While some authors reported detachment and anterior retraction of the diaphragm [88], others described incision and distraction of the diaphragm [73]. These variations appeared to be mostly dependent on the available retractor system.

In the middle lumbar spine, both retroperitoneal transpsoas and pre-psoas approaches via a far lateral or an anterolateral incision were commonly found. Most frequently, authors described the choice for either trans- or pre-psoas access routes based on the thickness of the psoas muscle in axial computed tomography or magnetic resonance images slices and the surgeons’ preference as well as the level affected. Due to the origin of the psoas muscle, a retroperitoneal pre-psoas approach may be easier in higher lumbar levels compared to the middle lumbar spine.

Another choice for the side of the approach refers to the coronary alignment of a potential deformity [63, 80]. Far lateral transpsoas approaches were rarely reported in lower lumbar spine corpectomies. In most cases, the iliac crest makes a strict lateral transpsoas approach challenging or even impossible. Moreover, splitting of the psoas muscle in the lower lumbar spine involves the risk for lumbosacral plexus injuries [79]. Therefore, L5 resections were commonly performed via a retroperitoneal oblique pre-psoas approach between vascular and muscle structures. Another option is to approach the lower lumbar spine through a strict anterior retroperitoneal approach with the advantage of a direct anterior visualization of the vertebras [15, 69]. In cases of extensive scar formation from previous abdominal surgeries, a transperitoneal access route was described by some authors [6, 83].

Assistance from an access surgeon may be helpful to mitigate the risk of surgical site morbidity in AL approaches [37]. Therefore, AL corpectomies may be more commonly performed in centers in which an access surgeon is readily available. However, whether the aid of an access surgeon is beneficial in reducing approach-related morbidity is still controversially discussed [34, 40, 72].

Overall, after anterior or lateral corpectomy procedures, a 4-point fixation directly adjacent to the resected vertebral body with lateral plates might not provide sufficient stability. Therefore, posterior instrumented fusion is commonly needed to gain additional stability [79].

Posterolateral approaches on the other hand are generally performed in a similar fashion throughout the entire lumbar spine. Approach variations are mostly dictated by the underlying pathology and the extent of bony resection required for adequate decompression and reconstruction [74]. However, the lumbar nerve roots may present a major obstacle in anterior column reconstruction following PL corpectomy [36]. Lumbar nerve routes exit the thecal sac in a rectangular trajectory close to the thoracolumbar junction and become more oblique towards the lumbosacral junction. Therefore, inserting a VBR device in the lower lumbar spine is described as less challenging, requires less nerve root and thecal sac retraction, and increases in complexity towards the upper lumbar spine. This is especially true for multilevel PL corpectomies [39, 79].

For both AL and PL corpectomies, minimally invasive variations have been described [1, 11, 11, 19, 21, 35, 53, 73]. However, due to the great heterogeneity of minimally invasive and less invasive variations, a detailed quantitative analysis was beyond the scope of the present review.

### Biomechanical considerations

A major point of controversy when comparing AL and PL approaches is the biomechanical stability after corpectomy. This is especially true regarding the choice of the VBR device as well as the need for an additional posterior approach for instrumentation after an AL corpectomy.

When performing a PL approach for corpectomy, a circumferential reconstruction including anterior column reconstruction as well as posterior bilateral pedicle screw instrumentation may be performed via the same approach. In AL corpectomies, however, several options exist. It is possible to perform unilateral instrumentation via a unilateral screw-plate or screw-rod construct via the same approach or to perform an additional posterior approach for bilateral pedicle screw instrumentation. In this review, it was found that the majority of articles reporting AL corpectomies described the use of an additional posterior approach for bilateral pedicle screw instrumentation. It was found, however, that overall implant-related complication rates were higher in PL corpectomies than in AL corpectomies regardless of the approach used for instrumentation. Clinical case series and biomechanical studies of the cervical spine have shown that especially in multilevel corpectomies, additional posterior instrumentation is required to ensure a biomechanically stable construct [31, 32]. Lumbar biomechanical studies, however, revealed that 60% of the segmental stability in the lumbar spine is provided by the anterior column [38]. This indicates the importance of appropriately sized and shaped VBR devices. In a cadaveric corpectomy model, round and wide rectangular footprint cages along with either unilateral plate or bilateral pedicle screw instrumentation were compared in six degrees of freedom range of motion test. The results of this study suggested that regardless of unilateral or bilateral instrumentation, wide rectangular footprint cages provided more stability if compared to cylindrical footprint cages. This indicates that the footprint of the cage had more impact on lumbar segmental stiffness than the mode of instrumentation [54]. Similar findings were reported in several other cadaveric studies indicating that rectangular footprint cages offer a more favorable axial loading distribution and hence more segmental stability than cages with a circular footprint [59, 60].

The fact that only AL approaches offer enough space to insert a wide rectangular footprint cage has been described as a potential benefit over PL approaches [73, 84]. Due to inconsistent reporting of the cage footprint shape used in AL approaches, it was not possible to quantitatively assess a clear correlation between cage footprint and implant-related complications in this review. However, in a retrospective study of 52 AL corpectomies in the thoracic and lumbar spine, Smith et al. [73] reported transitioning from cylindric to wide rectangular cages over the course of the study. Implant failure related to cage subsidence was observed in 13.5% of patients with cylindric footprint cages as opposed to 0% if a wide footprint cage was used.

### Outcomes and approach-related morbidity

Overall, the neurological improvement rates were similar in both groups. However, in the PL group, no neurological deterioration was reported, while in the AL group, deterioration occurred in 6.0% of patients.

The LOO was insignificantly higher for AL corpectomies. This may be attributable to the fact that the majority of articles reported the use of an additional posterior approach for instrumentation after AL corpectomy. Similarly, the mean EBL was higher in the AL group. In a study comparing thoracolumbar AL and PL approaches, it was observed that the EBL was only higher if additional posterior instrumentation was performed [49]. Overall, the complication and the revision rates were higher in the PL group than in the AL group. Since complete exposure of the thecal sac and the lumbar nerve roots is routinely required in PL corpectomies, accidental durotomies were far more common in PL corpectomies. In AL approaches, exposure of the dura is only required in cases of direct dural compression through bony fragments of the vertebral bodies. Moreover, achieving dural exposure over an AL approach is technically more challenging than in PL approaches, and not routinely accomplished.

In AL transpsoas approaches, the risk of lumbosacral plexus injury has been described as a major concern. Rates of postoperative thigh weakness after transpsoas surgery as high as 27% have been reported [14, 81]. For that reason, many spine surgeons advocate for the strict use of intraoperative neuromonitoring in AL transpsoas surgery in order to mitigate lumbosacral plexus damage [45, 46]. In a prospective multicentric study, dynamically evoked electromyography was utilized in 102 patients undergoing retroperitoneal transpsoas surgery. Postoperatively, 27.5% of patients experienced transient mild hip flexion weakness that resolved over 6 months in all patients. The authors concluded that rather than an actual nerve damage, a retraction-induced muscle trauma is responsible for postoperative lower extremity weakness after transpsoas surgery [81].

Overall, our results suggest higher approach-related morbidity in PL approaches for corpectomies in the lumbar spine. In a study comparing AL and PL approaches to the thoracolumbar spine, a similar morbidity rate of anterior-only and posterior-only approaches was found. Significantly increased morbidity was only described in cases of a combined anterior and posterior approach [49]. Similarly, a study reporting AL and PL corpectomies for neoplastic infiltration of the thoracic and thoracolumbar spine found no significant difference regarding complications or approach-related morbidity [85]. However, since these comparative studies report pooled outcomes of thoracic and lumbar corpectomies, the conclusions can be extrapolated to the lumbar spine only to a limited extent. When looking at the development in AL approaches over time, we observed a trend towards smaller accesses, wide footprint cages, and percutaneous posterior instrumentation in more recent studies. Recent technical advances such as neuronavigation, minimally invasive retractor systems, and modern cage devices might be responsible for the overall more favorable outcomes of AL corpectomies.

### Limitations

The major limitation of this study is the quality and quantity of available studies reporting on lumbar corpectomies. Most studies analyzed both thoracic and lumbar corpectomies together. Therefore, parameters such as EBL, LOO, and neurological outcome and complications could frequently not be included if they were not explicitly attributed to lumbar corpectomy patients.

Only 17 studies were focused exclusively on outcomes of lumbar corpectomies, and no study provided comparative data on AL versus PL lumbar corpectomies.

The majority of patient outcome data analyzed in this review originated from AL corpectomies (64.1%). Additionally, most of the studies included were case series without control groups as highlighted in Table 1. Due to the heterogeneity and inconsistency of the analyzed outcome parameters, a statistical analysis was only performed for a fraction of the included studies. The results of this review should therefore be interpreted as trends rather than statistical evidence.

## Conclusion

Both anterolateral and posterolateral lumbar corpectomies are challenging procedures with significant blood loss and high complication rates. While neurological outcomes of both approaches are similar, the results of this present review demonstrated significantly lower complication and revision rates in anterolateral corpectomies. However, especially outcomes of lumbar corpectomies via posterolateral approaches are scarcely reported, and to date, no comparative studies exist. This warrants further investigation by studies directly aimed at comparing outcomes between anterolateral and posterolateral corpectomies in the lumbar spine ideally including recent technological advancements like minimal-invasive approaches.

## Data Availability

Not applicable.

## Abbreviations

AL: Anterolateral
EBL: Estimated blood loss
LOO: Length of operation
PL: Posterolateral
VBR: Vertebral body replacement
